# {4,4′-Dichloro-2,2′-[2,2-dimethyl­propane-1,3-diylbis(nitrilo­methanyl­ylidene)]diphenolato-κ^4^ *O*,*N*,*N*′,*O*′}nickel(II)

**DOI:** 10.1107/S1600536811022732

**Published:** 2011-06-18

**Authors:** Hadi Kargar, Reza Kia, Elham Pahlavani, Muhammad Nawaz Tahir

**Affiliations:** a Chemistry Department, Payame Noor University, Tehran 19395-4697, I. R. of Iran; b X-ray Crystallography Laboratory, Plasma Physics Research Center, Science and Research Branch, Islamic Azad University, Tehran, Iran; c Department of Chemistry, Science and Research Branch, Islamic Azad University, Tehran, Iran; d Department of Physics, University of Sargodha, Punjab, Pakistan

## Abstract

In the title compound, [Ni(C_19_H_18_Cl_2_N_2_O_2_)], the Ni^II^ atom shows a slightly distorted square-planar geometry. The dihedral angle between the mean planes of the coordination rings is 9.15 (12)° while the dihedral angle between the mean planes of the two aromatic rings is 3.48 (16)°. In the crystal, pairs of inter­molecular C—H⋯O hydrogen bonds link neighboring mol­ecules into a chain along the *a* axis. The crystal structure is further stabilized by π–π inter­actions [centroid–centroid distance = 3.883 (2) Å].

## Related literature

For standard bond lengths, see: Allen *et al.* (1987 ▶). For background to Schiff base metal complexes, see: Granovski *et al.* (1993 ▶); Blower (1998 ▶); Elmali *et al.* (2000 ▶). For related structures, see: Fun *et al.* (2008 ▶); Kargar *et al.* (2008 ▶); Rayati *et al.* (2011 ▶).
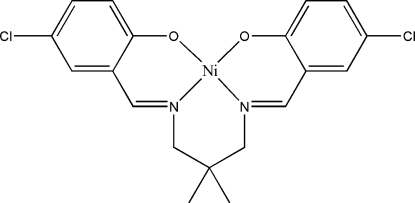


## Experimental

### 

#### Crystal data

[Ni(C_19_H_18_Cl_2_N_2_O_2_)]
*M* *_r_* = 435.96
Monoclinic, 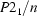

*a* = 6.9781 (3) Å
*b* = 23.2517 (11) Å
*c* = 11.8395 (5) Å
β = 105.828 (3)°
*V* = 1848.16 (14) Å^3^
*Z* = 4
Mo *K*α radiation
μ = 1.36 mm^−1^
*T* = 296 K
0.22 × 0.15 × 0.09 mm

#### Data collection

Bruker SMART APEXII CCD area-detector diffractometer
Absorption correction: multi-scan (*SADABS*; Bruker, 2005 ▶) *T*_min_ = 0.755, *T*_max_ = 0.888
14131 measured reflections
3354 independent reflections
2373 reflections with *I* > 2σ(*I*)
*R*_int_ = 0.062

#### Refinement

*R*[*F* ^2^ > 2σ(*F* ^2^)] = 0.039
*wR*(*F* ^2^) = 0.081
*S* = 1.02
3354 reflections
237 parameters
H-atom parameters constrained
Δρ_max_ = 0.31 e Å^−3^
Δρ_min_ = −0.29 e Å^−3^

### 

Data collection: *APEX2* (Bruker, 2005 ▶); cell refinement: *SAINT* (Bruker, 2005 ▶); data reduction: *SAINT*; program(s) used to solve structure: *SHELXTL* (Sheldrick, 2008 ▶); program(s) used to refine structure: *SHELXTL*; molecular graphics: *SHELXTL*; software used to prepare material for publication: *SHELXTL* and *PLATON* (Spek, 2009 ▶).

## Supplementary Material

Crystal structure: contains datablock(s) global, I. DOI: 10.1107/S1600536811022732/kj2181sup1.cif

Structure factors: contains datablock(s) I. DOI: 10.1107/S1600536811022732/kj2181Isup2.hkl

Additional supplementary materials:  crystallographic information; 3D view; checkCIF report

## Figures and Tables

**Table 1:** Hydrogen-bond geometry (Å, °)

*D*—H⋯*A*	*D*—H	H⋯*A*	*D*⋯*A*	*D*—H⋯*A*
C8—H8*A*⋯O1^i^	0.97	2.44	3.266 (4)	143
C12—H12*A*⋯O2^ii^	0.97	2.49	3.346 (4)	148

Symmetry codes: (i) 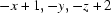; (ii) 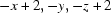.

**Fig. 1.**
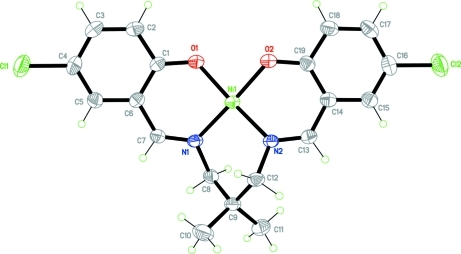
The molecular structure of the title compound, showing 50% probability displacement ellipsoids and the atomic numbering.

**Fig. 2.**
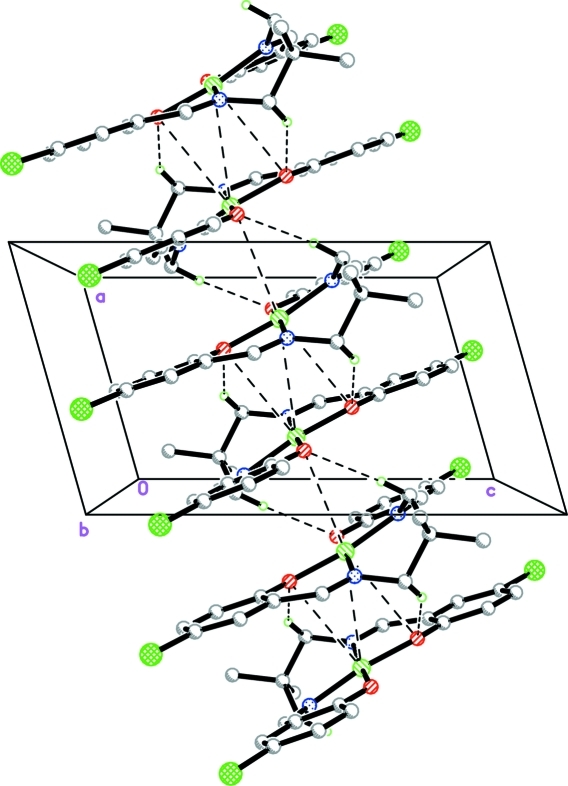
Packing diagram of the title compound, showing a 1-D infinite chain along the a-axis by the intermolecular C—H···O interactions. The intermolecular interactions are shown as dashed lines. Only the H atoms involved in hydrogen bonding are shown.

## supplementary crystallographic information

### Comment

Schiff base complexes are one of the most important stereochemical models in transition metal coordination chemistry, with their ease of preparation and structural variations (Granovski *et al.,* 1993). Metal derivatives of the Schiff bases have been studied extensively, and Ni^II^ and Cu(II) complexes play a major role in both synthetic and structurel research (Elmali *et al.,* 2000; Blower *et al.,* 1998). In continuation of our work on Schiff base ligands and their metal complexes (Fun *et al.*, 2008; Kargar *et al.*, 2008), we determined the X-ray crystal structure of the title compound.

The asymmetric unit of the title compound, Fig. 1, comprises one unit of the Schiff base complex. The bond lengths (Allen *et al.*, 1987) and angles are within the normal ranges and are comparable to the previous structures (Kargar *et al.*, 2008; Rayati *et al.*, 2011). The geometry ground the Ni^II^ atom is a sligthly distorted square-planar which is coordinated by the N_2_O_2_ donor atoms of the desired potentially tetradenate Schiff base ligand. The dihedral angle between the mean planes of the coordination plane rings, O1–Ni1–N1 and O2–Ni1–N2, is 9.15 (12)°. The dihedral angle between the  mean planes of the two aromatic rings is 3.48 (16)°.

The crystal structure is stabilized by pairs of intermolecular C—H···O hydrogen bonds which link neighboring molecules into a chain along the *a*-axis (Table 1, Fig. 2) and by π–π interactions [*Cg1*···*Cg2*^ii^ = 3.883 (2) Å; *Cg*1 and *Cg*2 are the centroids of the C1–C6 and C14–C19 benzene rings].

### Experimental

The title compound was synthesized by adding bis(5-chlorosalicylaldiminato)-2,3-propanediamine (2 mmol) to a solution of NiCl_2_. 6 H_2_O (2 mmol) in ethanol (30 ml). The mixture was refluxed with stirring for half an hour. The resultant red solution was filtered. Red single crystals of the title compound suitable for *X*-ray structure determination were recrystallized from ethanol by slow evaporation of the solvents at room temperature over several days.

### Refinement

All hydrogen atoms were positioned geometrically with C—H = 0.93–0.97 Å and included in a riding model approximation with U_iso_ (H) = 1.2 or 1.5 U_eq_ (C).

### Crystal data

**Table:**

[Ni(C_19_H_18_Cl_2_N_2_O_2_)]	*F*(000) = 896
*M**_r_* = 435.96	*D*_x_ = 1.567 Mg m^−^^3^
Monoclinic, *P*2_1_/*n*	Mo *K*α radiation, λ = 0.71073 Å
Hall symbol: -P 2yn	Cell parameters from 2545 reflections
*a* = 6.9781 (3) Å	θ = 2.5–27.4°
*b* = 23.2517 (11) Å	µ = 1.36 mm^−^^1^
*c* = 11.8395 (5) Å	*T* = 296 K
β = 105.828 (3)°	Block, red
*V* = 1848.16 (14) Å^3^	0.22 × 0.15 × 0.09 mm
*Z* = 4	

### Data collection

**Table:**

Bruker SMART APEXII CCD area-detector diffractometer	3354 independent reflections
Radiation source: fine-focus sealed tube	2373 reflections with *I* > 2σ(*I*)
graphite	*R*_int_ = 0.062
φ and ω scans	θ_max_ = 25.3°, θ_min_ = 2.5°
Absorption correction: multi-scan (*SADABS*; Bruker, 2005)	*h* = −8→8
*T*_min_ = 0.755, *T*_max_ = 0.888	*k* = −27→27
14131 measured reflections	*l* = −14→14

### Refinement

**Table:**

Refinement on *F*^2^	Primary atom site location: structure-invariant direct methods
Least-squares matrix: full	Secondary atom site location: difference Fourier map
*R*[*F*^2^ > 2σ(*F*^2^)] = 0.039	Hydrogen site location: inferred from neighbouring sites
*wR*(*F*^2^) = 0.081	H-atom parameters constrained
*S* = 1.02	*w* = 1/[σ^2^(*F*_o_^2^) + (0.024*P*)^2^ + 0.6017*P*] where *P* = (*F*_o_^2^ + 2*F*_c_^2^)/3
3354 reflections	(Δ/σ)_max_ = 0.001
237 parameters	Δρ_max_ = 0.31 e Å^−^^3^
0 restraints	Δρ_min_ = −0.29 e Å^−^^3^

### Special details

**Table:**

Geometry. All esds (except the esd in the dihedral angle between two l.s. planes) are estimated using the full covariance matrix. The cell esds are taken into account individually in the estimation of esds in distances, angles and torsion angles; correlations between esds in cell parameters are only used when they are defined by crystal symmetry. An approximate (isotropic) treatment of cell esds is used for estimating esds involving l.s. planes.
Refinement. Refinement of F^2^ against ALL reflections. The weighted R-factor wR and goodness of fit S are based on F^2^, conventional R-factors R are based on F, with F set to zero for negative F^2^. The threshold expression of F^2^ > 2sigma(F^2^) is used only for calculating R-factors(gt) etc. and is not relevant to the choice of reflections for refinement. R-factors based on F^2^ are statistically about twice as large as those based on F, and R- factors based on ALL data will be even larger.

### Fractional atomic coordinates and isotropic or equivalent isotropic displacement parameters (Å^2^)

**Table:**

	*x*	*y*	*z*	*U*_iso_*/*U*_eq_	
C1	0.6314 (4)	−0.09890 (13)	0.9031 (3)	0.0331 (7)	
C2	0.6332 (5)	−0.15815 (14)	0.9279 (3)	0.0418 (8)	
H2	0.6782	−0.1704	1.0054	0.050*	
C3	0.5704 (5)	−0.19826 (14)	0.8410 (3)	0.0445 (9)	
H3	0.5757	−0.2372	0.8594	0.053*	
C4	0.4986 (5)	−0.18048 (14)	0.7248 (3)	0.0437 (9)	
C5	0.4879 (5)	−0.12370 (14)	0.6975 (3)	0.0391 (8)	
H5	0.4359	−0.1122	0.6199	0.047*	
C6	0.5545 (4)	−0.08216 (13)	0.7852 (3)	0.0325 (7)	
C7	0.5194 (4)	−0.02263 (13)	0.7560 (3)	0.0352 (8)	
H7	0.4456	−0.0141	0.6798	0.042*	
C8	0.4941 (4)	0.07694 (13)	0.7879 (3)	0.0374 (8)	
H8A	0.4334	0.0915	0.8468	0.045*	
H8B	0.3897	0.0728	0.7151	0.045*	
C9	0.6463 (5)	0.12107 (13)	0.7696 (3)	0.0373 (8)	
C10	0.6756 (5)	0.11497 (16)	0.6468 (3)	0.0580 (11)	
H10A	0.7827	0.1395	0.6401	0.087*	
H10B	0.5554	0.1259	0.5892	0.087*	
H10C	0.7071	0.0757	0.6341	0.087*	
C11	0.5692 (5)	0.18114 (14)	0.7853 (3)	0.0535 (10)	
H11A	0.5712	0.1867	0.8659	0.080*	
H11B	0.4353	0.1851	0.7364	0.080*	
H11C	0.6527	0.2094	0.7632	0.080*	
C12	0.8481 (5)	0.11072 (14)	0.8595 (3)	0.0378 (8)	
H12A	0.9217	0.0823	0.8283	0.045*	
H12B	0.9240	0.1462	0.8704	0.045*	
C13	0.8949 (4)	0.12409 (13)	1.0628 (3)	0.0351 (8)	
H13	0.9212	0.1621	1.0476	0.042*	
C14	0.9299 (4)	0.10732 (14)	1.1837 (3)	0.0338 (8)	
C15	0.9916 (4)	0.14918 (15)	1.2715 (3)	0.0402 (8)	
H15	0.9932	0.1877	1.2509	0.048*	
C16	1.0493 (5)	0.13369 (16)	1.3869 (3)	0.0453 (9)	
C17	1.0546 (5)	0.07581 (17)	1.4183 (3)	0.0490 (9)	
H17	1.0970	0.0654	1.4970	0.059*	
C18	0.9979 (5)	0.03433 (15)	1.3341 (3)	0.0450 (9)	
H18	1.0042	−0.0041	1.3565	0.054*	
C19	0.9299 (4)	0.04858 (14)	1.2136 (3)	0.0352 (8)	
Ni1	0.74438 (6)	0.014738 (17)	0.97955 (3)	0.03310 (13)	
N1	0.5800 (4)	0.02007 (11)	0.8250 (2)	0.0333 (6)	
N2	0.8306 (4)	0.09081 (11)	0.9745 (2)	0.0316 (6)	
O1	0.6943 (3)	−0.06300 (9)	0.98952 (18)	0.0407 (6)	
O2	0.8753 (3)	0.00713 (9)	1.13730 (19)	0.0426 (6)	
Cl1	0.41277 (16)	−0.23188 (4)	0.61498 (9)	0.0684 (3)	
Cl2	1.12556 (16)	0.18517 (5)	1.49697 (9)	0.0697 (3)	

### Atomic displacement parameters (Å^2^)

**Table:**

	*U*^11^	*U*^22^	*U*^33^	*U*^12^	*U*^13^	*U*^23^
C1	0.0326 (18)	0.0283 (18)	0.040 (2)	0.0005 (15)	0.0131 (15)	0.0023 (16)
C2	0.047 (2)	0.037 (2)	0.044 (2)	0.0008 (17)	0.0160 (17)	0.0098 (17)
C3	0.046 (2)	0.028 (2)	0.061 (3)	0.0006 (16)	0.0182 (18)	0.0039 (18)
C4	0.040 (2)	0.033 (2)	0.056 (2)	−0.0042 (16)	0.0111 (17)	−0.0100 (18)
C5	0.039 (2)	0.040 (2)	0.038 (2)	0.0021 (16)	0.0085 (15)	0.0009 (16)
C6	0.0307 (18)	0.0297 (18)	0.039 (2)	−0.0019 (15)	0.0129 (15)	0.0005 (15)
C7	0.0370 (19)	0.037 (2)	0.0315 (18)	−0.0003 (16)	0.0098 (14)	0.0040 (15)
C8	0.0385 (19)	0.035 (2)	0.0336 (19)	0.0068 (16)	0.0021 (15)	0.0027 (15)
C9	0.048 (2)	0.033 (2)	0.0322 (19)	0.0043 (16)	0.0130 (16)	0.0067 (15)
C10	0.070 (3)	0.065 (3)	0.040 (2)	0.002 (2)	0.0174 (19)	0.011 (2)
C11	0.063 (3)	0.037 (2)	0.056 (2)	0.0097 (19)	0.0098 (19)	0.0074 (18)
C12	0.048 (2)	0.034 (2)	0.0355 (19)	−0.0008 (16)	0.0196 (16)	0.0063 (15)
C13	0.0365 (19)	0.0305 (19)	0.039 (2)	0.0028 (15)	0.0108 (15)	0.0045 (16)
C14	0.0273 (17)	0.038 (2)	0.0367 (19)	0.0046 (15)	0.0089 (14)	0.0038 (16)
C15	0.038 (2)	0.042 (2)	0.040 (2)	0.0016 (17)	0.0097 (16)	0.0020 (17)
C16	0.038 (2)	0.057 (3)	0.040 (2)	−0.0024 (18)	0.0091 (16)	−0.0106 (18)
C17	0.048 (2)	0.067 (3)	0.031 (2)	−0.003 (2)	0.0074 (16)	0.0037 (19)
C18	0.048 (2)	0.047 (2)	0.039 (2)	0.0015 (18)	0.0094 (16)	0.0102 (18)
C19	0.0294 (18)	0.042 (2)	0.0334 (19)	−0.0013 (16)	0.0064 (14)	0.0018 (16)
Ni1	0.0394 (2)	0.0284 (2)	0.0311 (2)	0.0014 (2)	0.00896 (17)	0.00520 (19)
N1	0.0383 (15)	0.0279 (15)	0.0336 (15)	0.0024 (13)	0.0094 (12)	0.0069 (12)
N2	0.0363 (15)	0.0295 (15)	0.0292 (15)	0.0029 (12)	0.0095 (12)	0.0054 (12)
O1	0.0572 (15)	0.0327 (13)	0.0316 (13)	−0.0017 (11)	0.0111 (11)	0.0057 (11)
O2	0.0556 (15)	0.0330 (14)	0.0354 (13)	0.0010 (11)	0.0061 (10)	0.0073 (11)
Cl1	0.0813 (8)	0.0419 (6)	0.0727 (7)	−0.0029 (5)	0.0050 (6)	−0.0173 (5)
Cl2	0.0797 (8)	0.0730 (8)	0.0478 (6)	−0.0004 (6)	0.0027 (5)	−0.0190 (5)

### Geometric parameters (Å, °)

**Table:**

C1—O1	1.301 (3)	C11—H11A	0.9600
C1—C6	1.407 (4)	C11—H11B	0.9600
C1—C2	1.408 (4)	C11—H11C	0.9600
C2—C3	1.369 (4)	C12—N2	1.474 (3)
C2—H2	0.9300	C12—H12A	0.9700
C3—C4	1.392 (5)	C12—H12B	0.9700
C3—H3	0.9300	C13—N2	1.279 (4)
C4—C5	1.357 (4)	C13—C14	1.440 (4)
C4—Cl1	1.748 (3)	C13—H13	0.9300
C5—C6	1.401 (4)	C14—C15	1.403 (4)
C5—H5	0.9300	C14—C19	1.411 (4)
C6—C7	1.431 (4)	C15—C16	1.363 (4)
C7—N1	1.282 (4)	C15—H15	0.9300
C7—H7	0.9300	C16—C17	1.394 (5)
C8—N1	1.469 (4)	C16—Cl2	1.742 (3)
C8—C9	1.534 (4)	C17—C18	1.366 (4)
C8—H8A	0.9700	C17—H17	0.9300
C8—H8B	0.9700	C18—C19	1.414 (4)
C9—C11	1.526 (4)	C18—H18	0.9300
C9—C10	1.529 (4)	C19—O2	1.305 (4)
C9—C12	1.535 (4)	Ni1—O2	1.850 (2)
C10—H10A	0.9600	Ni1—O1	1.851 (2)
C10—H10B	0.9600	Ni1—N2	1.874 (2)
C10—H10C	0.9600	Ni1—N1	1.880 (2)
			
O1—C1—C6	124.0 (3)	H11A—C11—H11C	109.5
O1—C1—C2	118.8 (3)	H11B—C11—H11C	109.5
C6—C1—C2	117.2 (3)	N2—C12—C9	113.5 (2)
C3—C2—C1	121.8 (3)	N2—C12—H12A	108.9
C3—C2—H2	119.1	C9—C12—H12A	108.9
C1—C2—H2	119.1	N2—C12—H12B	108.9
C2—C3—C4	119.7 (3)	C9—C12—H12B	108.9
C2—C3—H3	120.1	H12A—C12—H12B	107.7
C4—C3—H3	120.1	N2—C13—C14	125.1 (3)
C5—C4—C3	120.4 (3)	N2—C13—H13	117.4
C5—C4—Cl1	120.2 (3)	C14—C13—H13	117.4
C3—C4—Cl1	119.3 (3)	C15—C14—C19	120.4 (3)
C4—C5—C6	120.6 (3)	C15—C14—C13	118.9 (3)
C4—C5—H5	119.7	C19—C14—C13	120.0 (3)
C6—C5—H5	119.7	C16—C15—C14	120.4 (3)
C5—C6—C1	120.3 (3)	C16—C15—H15	119.8
C5—C6—C7	119.2 (3)	C14—C15—H15	119.8
C1—C6—C7	120.0 (3)	C15—C16—C17	120.1 (3)
N1—C7—C6	126.2 (3)	C15—C16—Cl2	121.0 (3)
N1—C7—H7	116.9	C17—C16—Cl2	118.9 (3)
C6—C7—H7	116.9	C18—C17—C16	120.4 (3)
N1—C8—C9	113.8 (2)	C18—C17—H17	119.8
N1—C8—H8A	108.8	C16—C17—H17	119.8
C9—C8—H8A	108.8	C17—C18—C19	121.4 (3)
N1—C8—H8B	108.8	C17—C18—H18	119.3
C9—C8—H8B	108.8	C19—C18—H18	119.3
H8A—C8—H8B	107.7	O2—C19—C14	124.1 (3)
C11—C9—C10	110.1 (3)	O2—C19—C18	118.7 (3)
C11—C9—C8	108.3 (3)	C14—C19—C18	117.2 (3)
C10—C9—C8	110.5 (3)	O2—Ni1—O1	83.80 (9)
C11—C9—C12	110.3 (3)	O2—Ni1—N2	92.76 (10)
C10—C9—C12	108.0 (3)	O1—Ni1—N2	172.47 (10)
C8—C9—C12	109.7 (2)	O2—Ni1—N1	172.29 (10)
C9—C10—H10A	109.5	O1—Ni1—N1	92.91 (10)
C9—C10—H10B	109.5	N2—Ni1—N1	91.30 (11)
H10A—C10—H10B	109.5	C7—N1—C8	118.0 (3)
C9—C10—H10C	109.5	C7—N1—Ni1	125.2 (2)
H10A—C10—H10C	109.5	C8—N1—Ni1	116.2 (2)
H10B—C10—H10C	109.5	C13—N2—C12	117.7 (3)
C9—C11—H11A	109.5	C13—N2—Ni1	126.1 (2)
C9—C11—H11B	109.5	C12—N2—Ni1	115.7 (2)
H11A—C11—H11B	109.5	C1—O1—Ni1	127.3 (2)
C9—C11—H11C	109.5	C19—O2—Ni1	126.8 (2)
			
O1—C1—C2—C3	−179.5 (3)	C15—C14—C19—O2	−179.8 (3)
C6—C1—C2—C3	2.5 (4)	C13—C14—C19—O2	−9.0 (4)
C1—C2—C3—C4	−1.4 (5)	C15—C14—C19—C18	−1.3 (4)
C2—C3—C4—C5	−0.9 (5)	C13—C14—C19—C18	169.6 (3)
C2—C3—C4—Cl1	−178.3 (2)	C17—C18—C19—O2	−179.1 (3)
C3—C4—C5—C6	2.0 (5)	C17—C18—C19—C14	2.3 (5)
Cl1—C4—C5—C6	179.3 (2)	C6—C7—N1—C8	−167.1 (3)
C4—C5—C6—C1	−0.7 (5)	C6—C7—N1—Ni1	4.1 (4)
C4—C5—C6—C7	−172.5 (3)	C9—C8—N1—C7	−116.4 (3)
O1—C1—C6—C5	−179.3 (3)	C9—C8—N1—Ni1	71.7 (3)
C2—C1—C6—C5	−1.5 (4)	O1—Ni1—N1—C7	−17.0 (2)
O1—C1—C6—C7	−7.6 (4)	N2—Ni1—N1—C7	156.7 (2)
C2—C1—C6—C7	170.2 (3)	O1—Ni1—N1—C8	154.3 (2)
C5—C6—C7—N1	−176.3 (3)	N2—Ni1—N1—C8	−32.0 (2)
C1—C6—C7—N1	11.9 (5)	C14—C13—N2—C12	−165.8 (3)
N1—C8—C9—C11	−155.3 (3)	C14—C13—N2—Ni1	6.0 (4)
N1—C8—C9—C10	84.0 (3)	C9—C12—N2—C13	−114.6 (3)
N1—C8—C9—C12	−34.9 (3)	C9—C12—N2—Ni1	72.8 (3)
C11—C9—C12—N2	84.4 (3)	O2—Ni1—N2—C13	−19.4 (3)
C10—C9—C12—N2	−155.2 (3)	N1—Ni1—N2—C13	154.1 (3)
C8—C9—C12—N2	−34.8 (4)	O2—Ni1—N2—C12	152.6 (2)
N2—C13—C14—C15	−176.9 (3)	N1—Ni1—N2—C12	−34.0 (2)
N2—C13—C14—C19	12.1 (5)	C6—C1—O1—Ni1	−12.5 (4)
C19—C14—C15—C16	−1.1 (4)	C2—C1—O1—Ni1	169.7 (2)
C13—C14—C15—C16	−172.0 (3)	O2—Ni1—O1—C1	−165.5 (2)
C14—C15—C16—C17	2.5 (5)	N1—Ni1—O1—C1	21.5 (2)
C14—C15—C16—Cl2	−179.8 (2)	C14—C19—O2—Ni1	−12.1 (4)
C15—C16—C17—C18	−1.5 (5)	C18—C19—O2—Ni1	169.4 (2)
Cl2—C16—C17—C18	−179.3 (3)	O1—Ni1—O2—C19	−164.2 (2)
C16—C17—C18—C19	−0.9 (5)	N2—Ni1—O2—C19	22.5 (2)

### Hydrogen-bond geometry (Å, °)

**Table:**

*D*—H···*A*	*D*—H	H···*A*	*D*···*A*	*D*—H···*A*
C8—H8A···O1^i^	0.97	2.44	3.266 (4)	143
C12—H12A···O2^ii^	0.97	2.49	3.346 (4)	148

Symmetry codes: (i) −*x*+1, −*y*, −*z*+2; (ii) −*x*+2, −*y*, −*z*+2.
